# Navigating a Dual Challenge: Management of Advanced Ovarian Cancer Diagnosed During the Second Trimester

**DOI:** 10.7759/cureus.96923

**Published:** 2025-11-15

**Authors:** Ik Hui Teo, Wan Nurul Ezyani Wan Jabarudin

**Affiliations:** 1 Obstetrics and Gynaecology, Universiti Malaya Medical Centre, Kuala Lumpur, MYS; 2 Obstetrics and Gynaecology, Universiti Malaya, Kuala Lumpur, MYS

**Keywords:** chemotherapy in pregnancy, high-risk pregnancy care, interval debulking surgery, neoadjuvant chemotherapy(nact), ovarian cancers, ovarian cancer second trimester, pregnancy cancer

## Abstract

Ovarian cancer during pregnancy is rare, and diagnosis is often delayed as symptoms overlap with normal pregnancy and tumour markers are unreliable. We present a case of a 31-year-old patient who presented at 21 weeks’ gestation with bilateral pleural effusions and respiratory compromise. Pleural cytology confirmed metastatic carcinoma consistent with a gynaecologic primary. After multidisciplinary review, neoadjuvant chemotherapy with carboplatin and paclitaxel was initiated in the second trimester and continued into the third trimester. Foetal surveillance revealed appropriate growth with no adverse effects. Labour was induced at 37 weeks, resulting in vaginal delivery of a healthy infant. The patient subsequently underwent interval cytoreductive surgery three weeks postpartum, followed by adjuvant chemotherapy, and remains in remission. This case demonstrates that platinum-taxane chemotherapy can be safely administered in pregnancy with favourable maternal and neonatal outcomes.

## Introduction

Ovarian cancer is the second most common gynaecologic malignancy diagnosed in pregnancy, with high-grade serous carcinoma (HGSC) being the predominant epithelial subtype [1]. Increasing use of routine antenatal ultrasonography has led to more frequent incidental detection of adnexal masses during pregnancy. Management poses unique challenges as both maternal and foetal outcomes must be considered.

In the first trimester, staging or cytoreductive surgery, with or without termination of pregnancy, remains the standard approach, depending on tumour histology, stage, prognosis, and patient preference [2]. Beyond 22 weeks, termination is generally less favourable due to advanced gestation and increased surgical morbidity. In such cases, chemotherapy may be employed as a temporising measure until term, with definitive cytoreduction performed in the postpartum period.

Chemotherapy is contraindicated in the first trimester due to teratogenicity. However, platinum-based regimens combined with taxanes are considered safe in the second and third trimesters, with no clear evidence of adverse maternal or foetal outcomes [3-5].

In this case, after stabilisation of respiratory compromise secondary to bilateral parapneumonic effusion, the patient was managed with antenatal chemotherapy until term. Foetal assessments, including detailed anomaly and serial growth scans, were unremarkable. She subsequently underwent interval debulking surgery one month postpartum. This outcome illustrates that adherence to standard oncologic principles, with modifications for gestational age, can achieve favourable maternal and foetal results.

## Case presentation

A woman in her 30s, gravida 3 para 2, with no significant past medical history, presented to the Emergency Department at 21 + 2 weeks’ gestation with worsening shortness of breath, orthopnoea, and a productive cough for the past two weeks. Her symptoms began about a month earlier with a runny nose and non-productive cough, for which she was prescribed oral antibiotics but did not complete the course. Over time, her cough became productive and was accompanied by right-sided pleuritic chest pain and progressive dyspnoea.

She initially consulted a general practitioner, was treated for costochondritis, and discharged with analgesics. Two weeks before this presentation, she returned to the Emergency Department but was discharged with unremarkable findings. She denied haemoptysis, weight loss, abdominal pain, diarrhoea, nausea, vomiting, arthralgia, or rash. The pregnancy had been uncomplicated to date. She reported no tobacco, alcohol, or recreational drug use. She worked in the Dietetics Unit of a local hospital and denied any sick contacts or recent travel.

**Figure 1:**
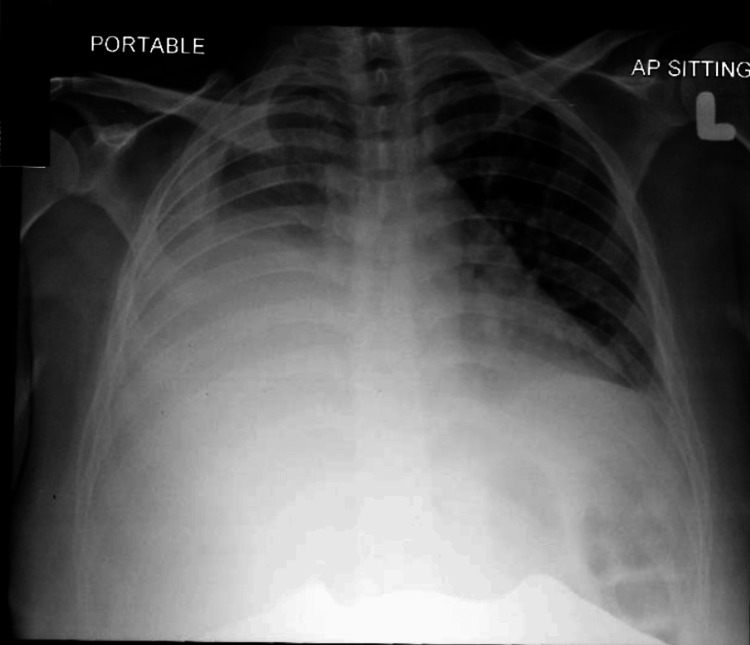
The chest X-ray which showed massive right-sided pleural effusion

She presented to the district hospital in respiratory distress. On arrival, she was tachypnoeic, with a respiratory rate of 38-40 breaths/minute. Her condition deteriorated, and a massive right-sided pleural effusion was identified, necessitating urgent thoracocentesis. Chest radiography confirmed a large effusion extending to the mid-zone. She was intubated and subsequently transferred to a tertiary centre (Figure 1).

She was treated for severe community-acquired pneumonia, with malignancy considered unlikely given her atypical presentation. She was admitted to the Intensive Care Unit and commenced on intravenous ampicillin-sulbactam and azithromycin. Foetal assessment was reassuring, with an estimated foetal weight of 486 g. In anticipation of possible preterm delivery, a course of corticosteroids for foetal lung maturation was administered.

She was extubated on the third hospital day but required re-intubation on day five after desaturation to 68% on room air. She developed left pleural effusion, and left-sided thoracocentesis was performed. Antibiotics were escalated to intravenous piperacillin-tazobactam. She was extubated the following day. The initial right-sided thoracocentesis drained 1,350 mL of haemorrhagic fluid, which was exudative; bacteriological cultures of sputum, blood, and pleural fluid were all non-conclusive. 

Further imaging was obtained for diagnostic clarification. MRI of the abdomen and pelvis (2 December 2024) demonstrated normal bilateral ovaries with no obvious pelvic mass, minimal ascites in the iliac fossae and subhepatic regions, mild bilateral pleural effusions with septations, and no peritoneal deposits or omental caking. Bilateral mild hydronephrosis and proximal hydroureter were also noted.

**Figure 2:**
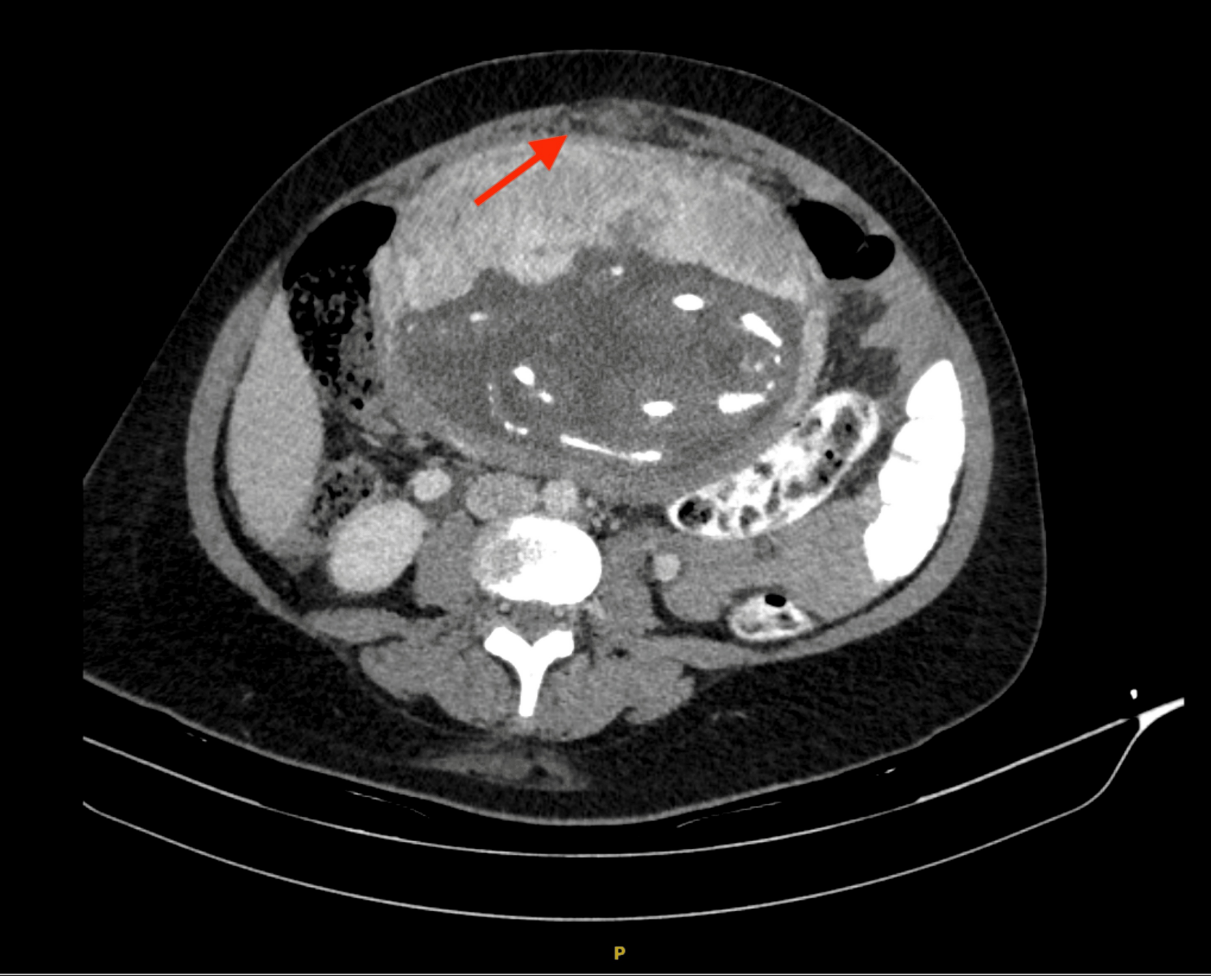
The initial CT scan demonstrated evidence of omental thickening, and the foetus within the uterus was visualised

Subsequently, a CT thorax, abdomen, and pelvis (4 December 2024) revealed a 2.6 × 2.3 × 2.3 cm cystic lesion with an enhancing wall at the left adnexa adjacent to the cervix, and a 5.3 × 3.4 × 3.0 cm soft tissue mass between the cervix and upper rectum, indenting the anterior rectal wall. Minimal ascites and pelvic peritoneal thickening were noted, without definite peritoneal deposits or enlarged abdominal/pelvic lymph nodes (Figure 2).

There was reaccumulation of pleural effusion with right lower lobe pleural thickening and sub-centimetre mediastinal and right axillary lymphadenopathy. This raises suspicion of ovarian malignancy.

Two weeks into admission, pleural fluid cytology confirmed metastatic carcinoma, suggestive of a primary tumour from the female genital tract. Immunohistochemistry was positive for BER-EP4, CK7, and PAX8 and negative for CK20, calretinin, TIF1, and Napsin A. Fine-needle aspiration of the right axillary lymph node also demonstrated metastatic carcinoma likely originating from the female genital tract. 

During the third week of admission, she experienced further desaturation due to reaccumulated pleural fluid, necessitating bilateral thoracic pigtail catheter insertion. Following multidisciplinary discussion, including gynaecologic oncology, radiology, pathology, thoracic surgery, and medical specialities, neoadjuvant chemotherapy was initiated. She received weekly carboplatin (AUC 2) and paclitaxel (60 mg/m², based on current weight) starting at 25 + 5 weeks, escalating to full-dose three-weekly cycles (carboplatin AUC 5, paclitaxel 175 mg/m², based on pre-pregnancy weight) for three further cycles. The final antenatal cycle was completed at 34 weeks’ gestation.

Serial ultrasound assessments at 21, 26, 28, 33, and 35 weeks showed normal foetal growth, amniotic fluid indices, and Doppler studies. At 32 weeks, a further multidisciplinary team (MDT) meeting concluded that, as she was multiparous with prior vaginal deliveries, induction of labour at term would be preferable to caesarean delivery with concurrent cytoreductive surgery, which would carry higher morbidity.

Labour was induced at 37 weeks with vaginal prostaglandins, and she delivered a healthy male infant weighing 2.54 kg at 37 + 2 weeks. The neonate required no respiratory or intensive care support. She was discharged on postpartum day three and prescribed cabergoline to suppress lactation.

**Figure 3:**
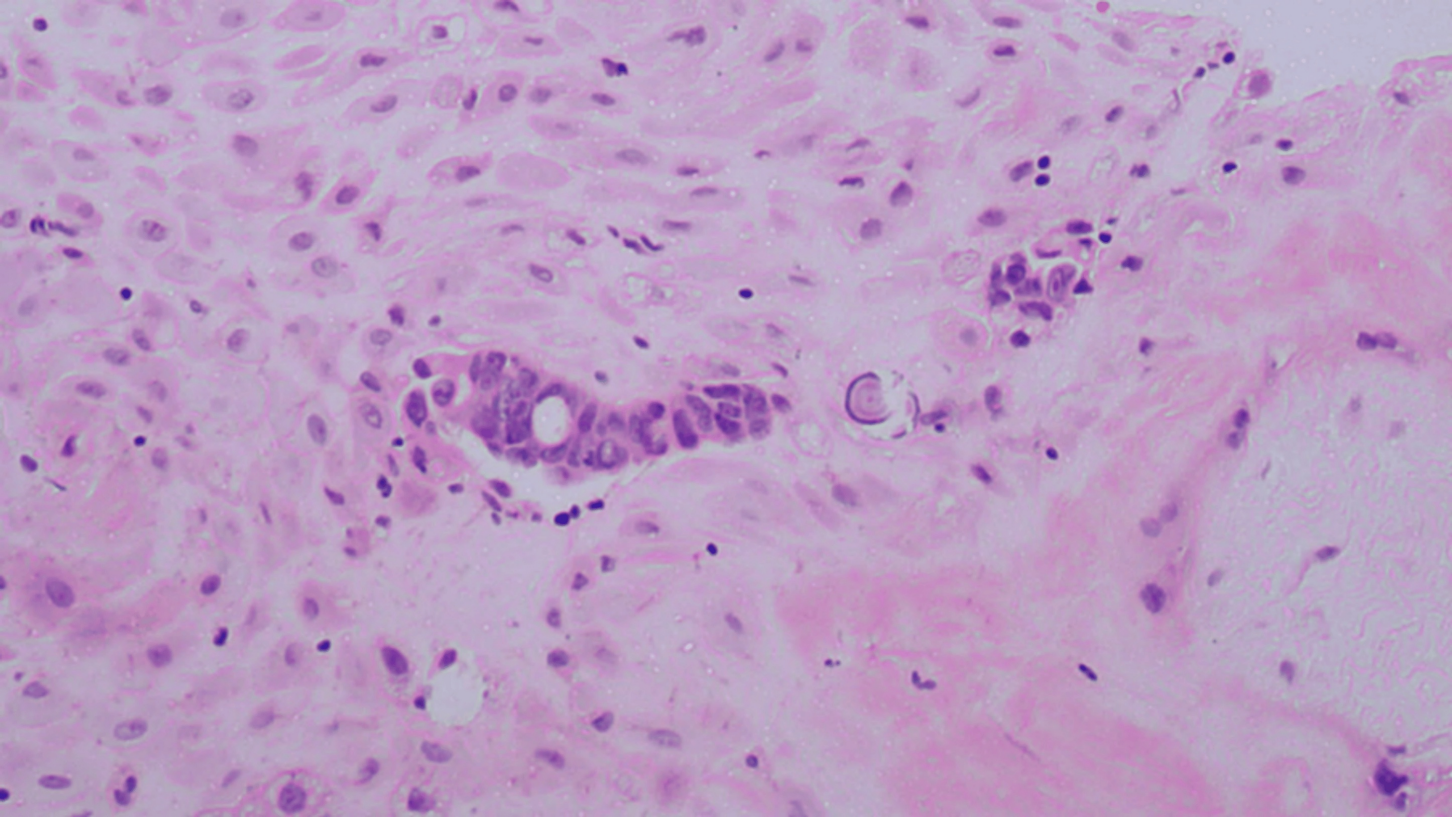
Cluster of metastatic tumour cells with adjacent psammoma body. The tumour cells have pleomorphic irregular hyperchromatic nuclei (H&E 40x) H&E: hematoxylin and eosin

**Figure 4:**
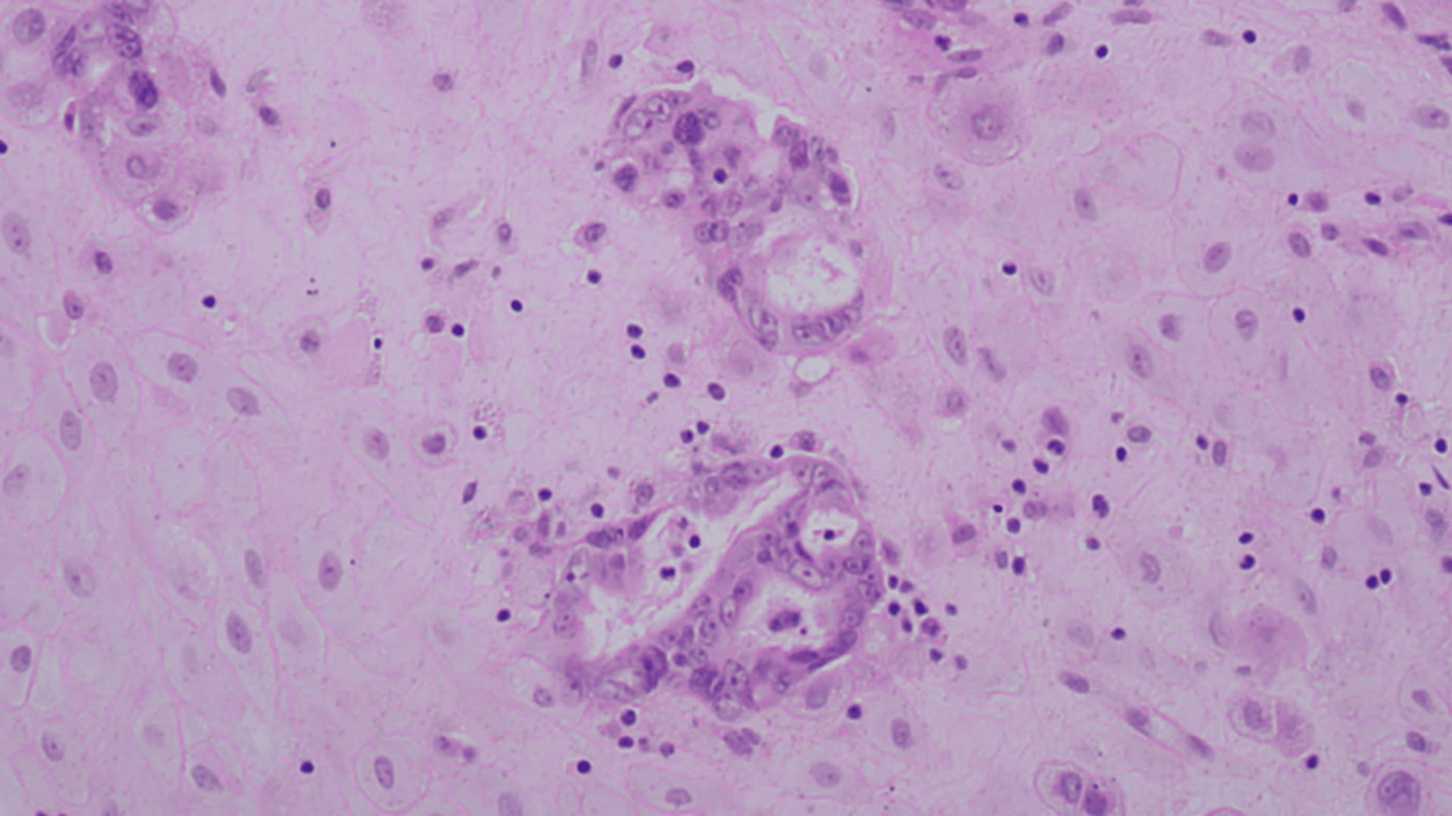
Mulitple clusters of metastatic tumour cells forming fused irregular abortive glands. The tumour cells have pleomorphic irregular vesicular nuclei with prominent nucleoli (H&E 40x) H&E: hematoxylin and eosin

**Figure 5:**
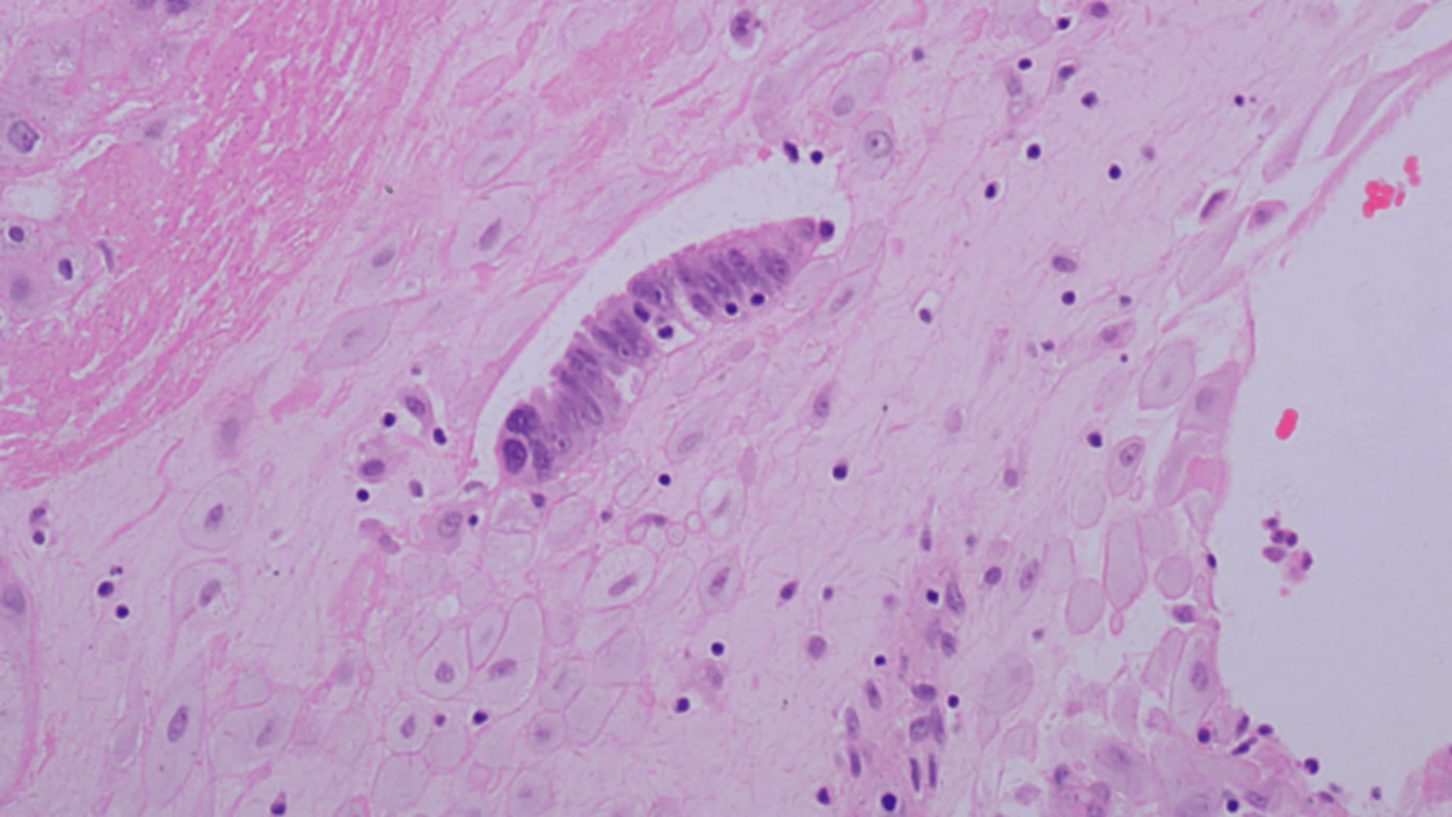
Cluster of metastatic tall columnar ciliated tumour cells with pleomorphic hyperchromatic nuclei

Histopathological examination of the placenta revealed clusters of atypical cells, some forming abortive and fused glands, within the foetal membranes. Most of the cells are poorly differentiated, with few retaining a columnar ciliated appearance. Occasional psammoma bodies are also seen. The chorionic villi were appropriate for gestational age, with no significant inflammation or vascular abnormality. The decidua and umbilical cord were unremarkable, and no malignant cells were identified in the cord or placental disc. Immunohistochemistry demonstrated strong and diffuse MNF-116 positivity, with negative staining for vimentin, CD68, Napsin A, and WT-1. Weak, equivocal cytoplasmic staining was seen for PAX-8 and β-hCG. These findings are consistent with metastatic carcinoma involving the placental tissue (Figures 3-5).

**Figure 6:**
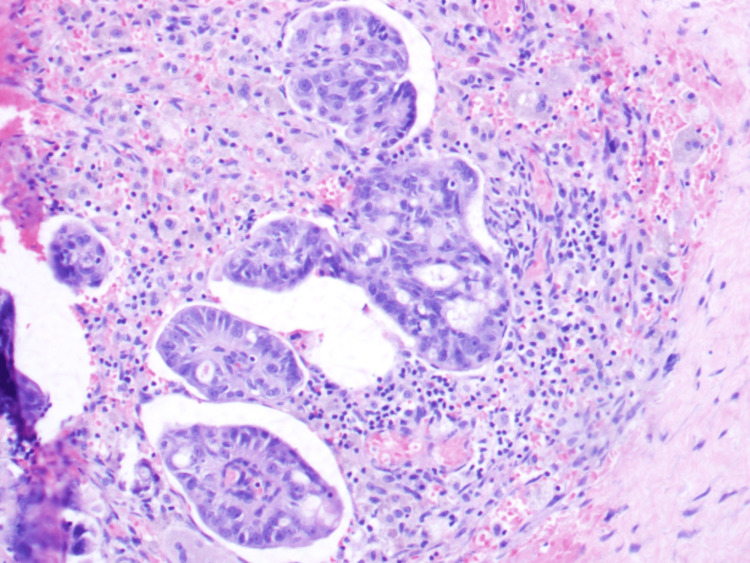
Clusters and small papillae of residual malignant cells with marked nuclear pleomorphism and hyperchromasia, with frequent mitosis and apoptotic bodies

**Figure 7:**
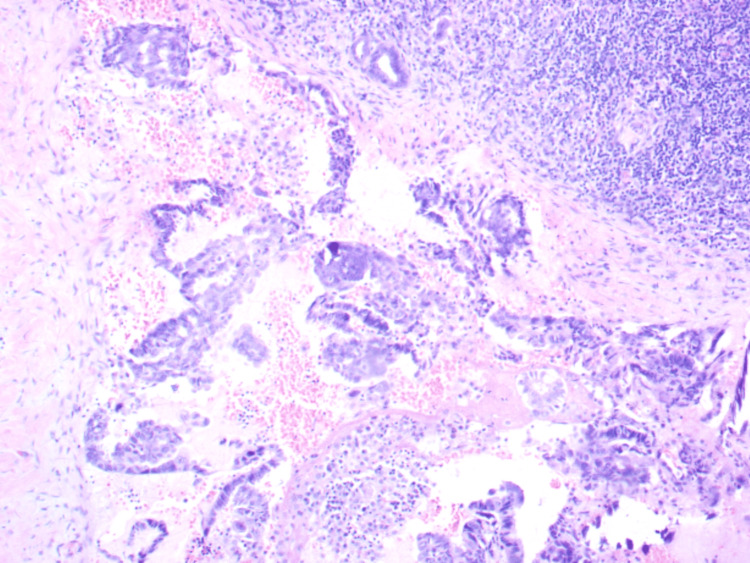
Metastatic high-grade serous carcinoma in lymph node, the malignant cells are in small papillae with marked nuclear pleomorphism

Chemotherapy was resumed three weeks postpartum with an additional three cycles of carboplatin and paclitaxel, which were well-tolerated without complications. Histopathological assessment of the cytoreductive surgery specimens confirmed residual high-grade serous carcinoma involving the left ovary, bilateral fallopian tubes, parametria, omentum, pelvic lymph nodes, and mesoappendix, with an overall chemotherapy response score of 2 (Figures 6-7). Germline testing subsequently revealed a pathogenic BRCA1 mutation.

**Figure 8:**
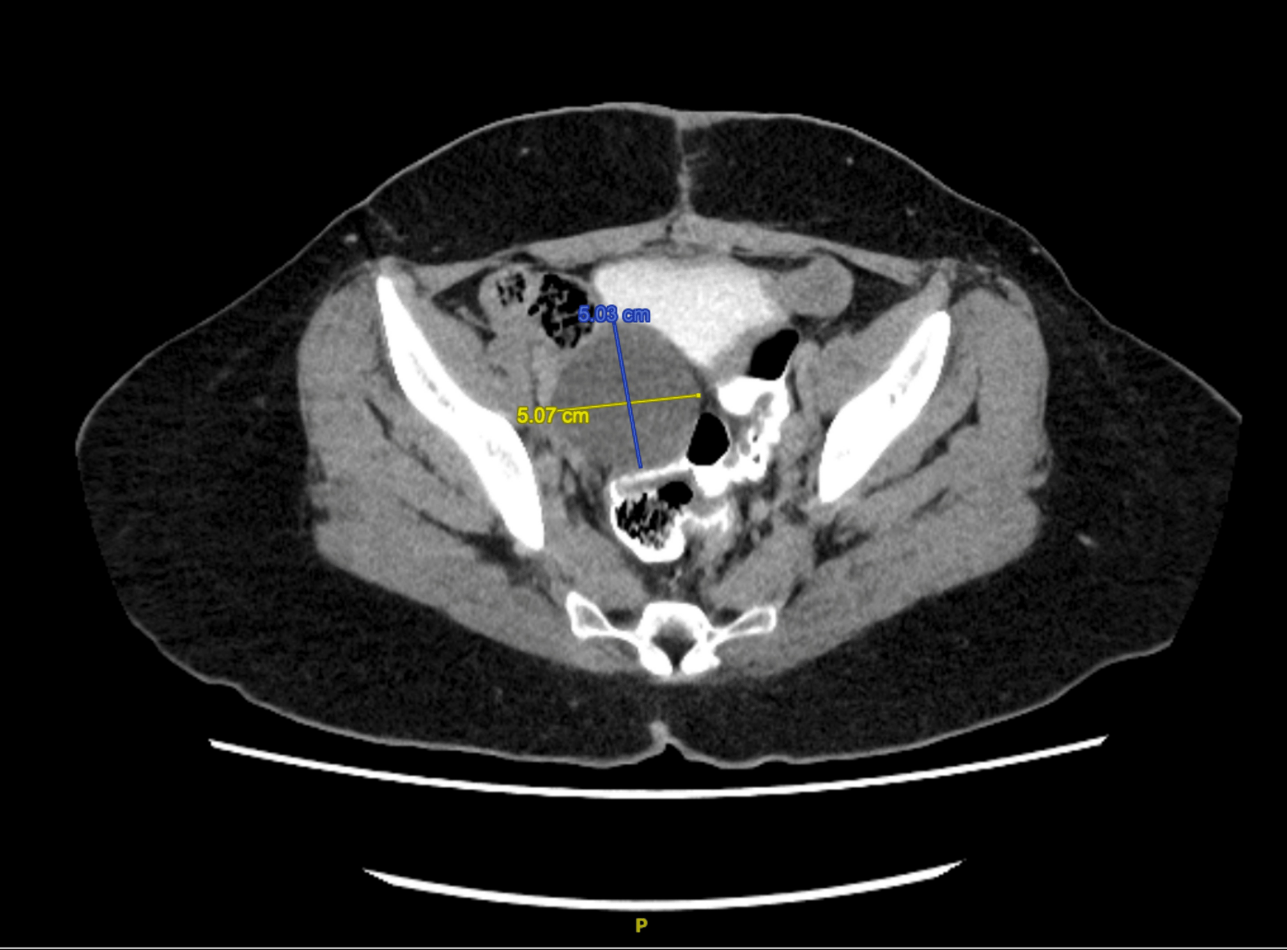
Postoperative imaging revealed a thin-walled cystic adnexal lesion in the right pelvis, most likely representing a lymphocoele

A repeat CT scan performed four weeks after her final chemotherapy demonstrated a new thin-walled right adnexal cystic lesion, most likely representing a postoperative lymphocoele, measuring approximately 5.0 × 4.9 × 5.2 cm (Figure 8). No other significant thoracic, abdominal, or pelvic abnormalities were noted. Her CA-125 level remains low at 6 U/ml, and she is otherwise well and asymptomatic.

Differential diagnosis

Throughout the course of managing this case, several differential diagnoses were considered. Initially, community-acquired pneumonia was suspected, given the patient’s recurrent respiratory symptoms and subsequent respiratory distress. Pulmonary tuberculosis was also considered when pleural effusion was identified; however, this was excluded with a negative MTB GeneXpert test. Following pleural cytology results suggestive of a primary malignancy from the female genital tract, chemotherapy was initiated, leading to a good clinical response and a favourable pregnancy outcome.

Outcome and follow-up

The patient responded well to chemotherapy, achieving stable oxygenation on room air and resuming daily activities after the first cycle. Her pregnancy progressed without complications, and she delivered a healthy male infant at 37 + 2 weeks following induction of labour. The intrapartum course was uneventful.

Postpartum, she completed the planned chemotherapy regimen and reported good functional recovery. While she requested counselling to help manage the emotional impact of chemotherapy and caring for a newborn, she successfully adapted and has since returned to her daily activities. She remains on medical leave in accordance with local regulations. On follow-up, she remains asymptomatic, with normal CA-125 levels and no clinical evidence of disease recurrence.

## Discussion

Ovarian cancer during pregnancy is a rare and challenging clinical scenario, with an incidence of approximately one in 10,000 to one in 100,000 pregnancies, depending on the population studied [5]. Adnexal masses are found in up to 3% of pregnancies, only 1-3% prove malignant, and the majority are germ cell tumours [6,7]. Ovarian cancer takes the sixth position among the most common malignancies affecting pregnant women, following breast cancer, thyroid cancer, cervical cancer, and Hodgkin lymphoma [8]. It is the second most common gynaecologic cancer diagnosed during pregnancy, complicating one in 15,000 to one in 32,000 pregnancies [9]. 

A literature search showed there were no case reports of ovarian cancer in pregnancy reported in Malaysia, and global literature remains limited to isolated case reports and small series [10]. Management is further complicated by the need to balance maternal oncologic outcomes with foetal safety, requiring individualised decision-making and multidisciplinary input. In 2024, the European Society for Medical Oncology (ESMO) published a guideline on gynaecologic cancers in pregnancy, which is based on the European Society of Gynaecological Oncology (ESGO)-ESMO- European Society of Pathology (ESP) consensus meeting [11,12]. This provides guidance for the management of this rare condition. 

HGSC of the ovary or fallopian tube, the most common epithelial subtype, is infrequently diagnosed during gestation and is often mistaken for benign adnexal pathology because of overlapping symptoms with normal pregnancy, such as abdominal discomfort, bloating, or dyspnoea. In our patient, the initial presentation with recurrent pleural effusions and respiratory compromise was atypical, delaying suspicion for malignancy. 

Diagnostic challenges are considerable. Tumour markers such as CA-125 are unreliable during pregnancy due to physiologic fluctuations [13,14]. The intervention of the adnexal masses is often delayed until the postpartum period, unless the patient is symptomatic or if the risk of malignancy or torsion is high [15].

Ultrasound is a cornerstone modality in obstetric imaging owing to its safety, diagnostic accuracy, cost-effectiveness, and widespread availability [16]. The sonographic appearance of adnexal masses in pregnancy is generally consistent with findings in non-pregnant patients [17]. Among the validated tools for malignancy risk assessment in pregnancy are the International Ovarian Tumour Analysis (IOTA) Simple Rules and the IOTA Assessment of Different NEoplasias in the adneXa (ADNEX) model. In a recent single-centre study involving 153 pregnant patients, including 12 malignancies, the IOTA Simple Rules demonstrated a sensitivity of 92% and specificity of 69% for malignancy prediction [18]. In contrast, the ADNEX model, incorporating patient age, serum CA-125 concentration, and presence of ascites, may be less applicable in pregnancy due to altered physiological baselines, potentially accounting for its comparatively lower predictive performance in this population [19].

Timing and modality of treatment are pivotal. According to the 2024 ESGO-ESMO-ESP consensus, cytoreductive surgery is preferred if feasible without undue foetal or maternal risk, but in advanced gestations, particularly beyond 22 weeks, delaying definitive surgery until postpartum and initiating neoadjuvant chemotherapy is a reasonable strategy [11,12]. Chemotherapy is contraindicated in the first trimester due to teratogenicity but can be administered relatively safely in the second and third trimesters [2]. Platinum-taxane combinations (e.g., carboplatin plus paclitaxel) remain the standard of care and are associated with acceptable foetal outcomes [4]. Our patient tolerated this regimen well, and foetal growth remained normal on serial ultrasounds

Published outcomes suggest that chemotherapy during pregnancy, when commenced after 14 weeks’ gestation, is not associated with increased rates of congenital malformations but may increase the risk of preterm delivery [20]. In our patient, the decision to proceed with induction of labour at term, followed by interval cytoreductive surgery, was made in consultation with the MDT. Given her history of prior vaginal delivery, her likelihood of achieving spontaneous vaginal birth was considered high. The foetal size was also deemed appropriate for vaginal delivery. This approach aimed to minimise the maternal morbidity associated with performing cytoreductive surgery during caesarean section, while still achieving optimal disease control. Labour was induced with a prostaglandin agent, and she subsequently delivered vaginally.

Psychosocial considerations must not be overlooked. Balancing the physical toll of chemotherapy with the demands of late pregnancy, childbirth, and early motherhood is emotionally taxing, as reflected in our patient’s account. Referral for psychological support and counselling should be a routine component of care in such cases. 

Learning points

Patients presenting with recurrent respiratory conditions should not be taken lightly, as these may indicate underlying serious issues. In cases where cancer is diagnosed during pregnancy, chemotherapy can be administered safely in line with standard management for non-pregnant adults, particularly when initiated after the first trimester. The involvement of an MDT is essential in managing such complex cases, especially during pregnancy, to ensure both maternal and foetal safety. Furthermore, decisions regarding care should be made collaboratively between the patient, their next-of-kin, and the managing medical professionals to achieve the best possible outcomes.

Patient's perspective

This is the experience recorded by the patient: "In my first trimester, I felt extremely lethargic. I could not stand for long periods and often needed to lie down most of the day. If I forced myself to keep working, I experienced spotting, which required medical leave. Thankfully, these symptoms improved once I entered the second trimester.

At that time, I also developed bloating and altered bowel habits, alternating between constipation and diarrhoea. After adjusting my diet and increasing fibre intake, this settled. Later in the second trimester, I started having presyncopal episodes and shortness of breath, especially on exertion. I also experienced intermittent chest pain and insomnia, which became most noticeable during prayers, particularly in the prostration position. Initially, I was told it might be muscular pain, but after several visits to the Emergency Department, pleural effusion was finally identified, and I was admitted to the ICU at a tertiary centre. The treatment was long and discouraging at first, but with improvement, my spirit became stronger.

After delivery, while continuing chemotherapy and caring for my newborn and other children, I often felt stressed and irritable. To cope, I turned to fasting, which helped me feel calmer and less prone to anger when I was exhausted. I was also referred for counselling, which I believe has greatly helped me manage stress and maintain a positive outlook."

## Conclusions

In conclusion, this case highlights the need to maintain a broad differential diagnosis in pregnant patients with unexplained pleural effusions or persistent respiratory symptoms and to involve an MDT early to balance maternal and foetal priorities. Adhering to evidence-based oncologic principles, while adapting treatment timing and modalities to the context of pregnancy and incorporating patient values, is essential to optimising both survival and quality of life. The favourable maternal and foetal outcomes in this case demonstrate that, with careful planning and coordinated multidisciplinary care, advanced-stage ovarian or fallopian tube carcinoma can be successfully managed during pregnancy without compromising oncologic standards.
